# Management of hospital beds and ventilators in the Gauteng province, South Africa, during the COVID-19 pandemic

**DOI:** 10.1371/journal.pgph.0001113

**Published:** 2022-11-02

**Authors:** Mahnaz Alavinejad, Bruce Mellado, Ali Asgary, Mduduzi Mbada, Thuso Mathaha, Benjamin Lieberman, Finn Stevenson, Nidhi Tripathi, Abhaya Kumar Swain, James Orbinski, Jianhong Wu, Jude Dzevela Kong

**Affiliations:** 1 Africa-Canada Artificial Intelligence and Data Innovation Consortium (ACADIC), Laboratory for Industrial and Applied Mathematics, York University, Toronto, Canada; 2 Africa-Canada Artificial Intelligence and Data Innovation Consortium (ACADIC), School of Physics, Institute for Collider Particle Physics, University of the Witwatersrand, Johannesburg, South Africa; 3 iThemba LABS, National Research Foundation, Cape Town, South Africa; 4 Africa-Canada Artificial Intelligence and Data Innovation Consortium (ACADIC), The Advanced Disaster, Emergency and Rapid Response Program, York University, Toronto, Canada; 5 Head of Policy at Gauteng Office of the Premier, Johannesburg, South Africa; 6 University of the Witwatersrand, Johannesburg, South Africa; 7 Africa-Canada Artificial Intelligence and Data Innovation Consortium (ACADIC), The Dahdaleh Institute for Global Health Research, York University, Toronto, Canada; Management Sciences for Health, UGANDA

## Abstract

We conducted an observational retrospective study on patients hospitalized with COVID-19, during March 05, 2020, to October 28, 2021, and developed an agent-based model to evaluate effectiveness of recommended healthcare resources (hospital beds and ventilators) management strategies during the COVID-19 pandemic in Gauteng, South Africa. We measured the effectiveness of these strategies by calculating the number of deaths prevented by implementing them. We observed differ ences between the epidemic waves. The length of hospital stay (LOS) during the third wave was lower than the first two waves. The median of the LOS was 6.73 days, 6.63 days and 6.78 days for the first, second and third wave, respectively. A combination of public and private sector provided hospital care to COVID-19 patients requiring ward and Intensive Care Units (ICU) beds. The private sector provided 88.4% of High care (HC)/ICU beds and 49.4% of ward beds, 73.9% and 51.4%, 71.8% and 58.3% during the first, second and third wave, respectively. Our simulation results showed that with a high maximum capacity, i.e., 10,000 general and isolation ward beds, 4,000 high care and ICU beds and 1,200 ventilators, increasing the resource capacity allocated to COVID- 19 patients by 25% was enough to maintain bed availability throughout the epidemic waves. With a medium resource capacity (8,500 general and isolation ward beds, 3,000 high care and ICU beds and 1,000 ventilators) a combination of resource management strategies and their timing and criteria were very effective in maintaining bed availability and therefore preventing excess deaths. With a low number of maximum available resources (7,000 general and isolation ward beds, 2,000 high care and ICU beds and 800 ventilators) and a severe epidemic wave, these strategies were effective in maintaining the bed availability and minimizing the number of excess deaths throughout the epidemic wave.

## 1 Introduction

COVID-19 has imposed and continues to impose challenges on many countries, worldwide. In the early stages of the pandemic the number of cases and hospitalizations were on a rapid rise, over- whelming the healthcare systems in several countries. In Italy, the number of cases and hospitalizations were rising exceptionally fast during the first wave leading to a strict nationwide lockdown. The number of cases increased from 3 to 59,138 between February 20 and March 22, 2020, and there were more than 4000 patients admitted to intensive care units (ICU) on April 1st, which is the highest value during the entire pandemic [1]. Spain faced a similar situation where the number of COVID cases exceeded 100,000 by April 1st, 2020. The country had its highest number of hospitalizations (over 30,000) on March 29, 2020 [1]. While the whole world has been greatly impacted by COVID-19, this impact has been higher on the low- and middle-income countries. More than five million deaths have been reported in the world during the pandemic with 65% of the deaths in low- and middle-income countries (Date accessed: November 08, 2021) [1]. The combination of a high burden of the disease and a low capacity of healthcare resources could result in a higher number of excess deaths.

On March 07, 2020, the world health organization published critical preparedness guidelines [2] and many countries put strict non-pharmaceutical interventions in place to keep the burden of the disease below the healthcare capacity. However, these interventions could only reduce the burden of the disease to a certain level due to several factors such as the evolution of SARS-CoV-2 virus to more transmissible variants with higher severity of the symptoms [3] as well as constraints affecting the interventions such as the economic consequences of a strict lockdown [4]. With the production and approval of vaccines against COVID-19, a combination of pharmaceutical and non-pharmaceutical interventions has been used to control the spread of the disease. While high-income countries have been able to vaccinate a major proportion of their population, low and middle-income countries struggle with both vaccinating the population and providing adequate care to COVID patients. To this date, 65% of the population in high-income countries have been fully vaccinated while only 36% of the population in low- and middle-income countries are fully vaccinated (Date accessed: November 08, 2021) [1]. Many countries in Africa are among those with the lowest rates of vaccination [1].

Available resources in healthcare systems vary across countries [5]. In case of the COVID-19 pandemic three types of resources are considered crucial for effective response to the pandemic: general hospital beds, intensive care unit (ICU) beds and ventilators [6]. One of the indicators of the World Health Organization’s Global Health Observatory is the number of hospital beds per 10,000 people [7]. According to the latest estimate, provided by Our World in Data for the number of hospital beds per 1000 population, the number varies between 0.1 and 13.8 [5]. Many high-income countries have between 2 to 4 hospital beds per 1000 population.

In South Africa (SA), there are 2.3 hospital beds per 1000 people, which is comparable to high-income countries such as Canada, Denmark, Sweden, and the United Kingdom, however the distribution of the hospital and particularly ICU beds is inhomogeneous in the country [8]. Healthcare facilities are managed by both public and private sectors in SA, with 75% of ICU beds provided by the private sector while 80% of the population rely on the public sector [9]. Unfortunately, a recent accurate estimate of the number of available resources is not available for SA. Based on an estimate given for 2008–2009, the number of ICU and high care (HC) beds available in the public and private sectors were 1186 and 3533, respectively [10]. An estimate of 5000 available ICU beds was given in the beginning of the pandemic out of which 2500 beds were allocated to COVID-19 patients.

Several methods have been used to study the increased demand for healthcare resources and the available resources. Capistran et al. [11] have used a dynamical and observational model to predict the healthcare demand for the second wave of the pandemic in Mexico. Barrett et al. [12] used an individual based simulation model to predict the number of days to resource depletion and the number of excess deaths due to resource depletion in Ontario, Canada. Irragori et al. [13] adapted the Ontario model to predict the healthcare resources demand for PCR tests and the resource depletion date in Valle del Cauca region in Colombia. Noronha et al. [14] estimated the healthcare demand for hospital resources (general ward, ICU beds and ventilators) and hospital occupancy rates during the COVID-19 pandemic for all regions in Brazil. Sjodin et al. [15] used a dynamical model to estimate the demand for ICU beds and the contributors to the excess deaths during COVID-19 in Sweden. In UK, Wood et al. [16] conducted a scenario analysis for the combination of increasing the healthcare resources and reducing the burden of the diseases, particularly the number of hospital admissions, using a stochastic discrete event simulation model.

Studies have been conducted for the countries in Africa. Barasa et al. [17] conducted a scenario analysis for the health system of Kenya. Denhard et al. [18] defined an index for oxygen treatment capacity and facility treatment capacity to assess the preparedness of the Mozambican health system to respond to COVID-19 pandemic. In Nigeria, Ogunbameru et al. [19] adapted the model used for Ontario, Canada, to estimate the healthcare resources depletion date and the corresponding number of excess deaths. For South Africa, studies have been conducted to discuss the spatial distribution of hospital beds across the country and the challenges of sharing the healthcare resources between provinces [8, 20]. The need for inter-facility collaboration for a better and a more effective health systems response to COVID-19 pandemic in South Africa was addressed by Naidoo et al. in [21]. Morrow et al. [9] discussed the ethical considerations of prioritizing critical care resources allocation to COVID-19 patients when the demand for healthcare resources exceeds the capacity.

Maintaining availability of healthcare resources during events where a large volume of patients needs hospital care such as during disasters and pandemics is challenging. Computer simulations can help determine more effective actions and make optimal decisions both prior to an emergency situation and during an ongoing emergency. In case of the COVID-19 pandemic actions and guidelines have been provided by organizations such as the World Health Organization [22] and governments. Some of the healthcare resources management challenges and guidelines provided by the U.S. Department of Health & Human Services [23] are:

1. Identifying non-emergency procedures such as elective surgeries or non-surgery procedures and canceling or postponing them
2. Increasing the bed capacity via different actions such as opening closed units or converting single rooms to double
3. Identifying patients for early discharge and providing follow up services
4. Using non-patient care areas in the facilities
5. Providing hospital bed data from and between all facilities
6. Transferring patients between facilities
7. creating alternate care sites

The purpose of this study is to examine the healthcare demand during the pandemic and the effectiveness of different management strategies as well as the association of the increased healthcare demand with healthcare resource depletion and its consequences (e.g., number of excess deaths due to resource scarcity). Through this study, we aim to evaluate how the combination of public and private sectors has worked in providing adequate care to patients during COVID-19. While agent-based dynamical models have been used in a number of the studies mentioned above, the model provided here is developed based on the characteristics of the data available in the Gauteng province and therefore it has a structure that differs from other studies (note that Gauteng is the most populated province in South Africa that generates more than a third of South Africa’s GDP [24]). The purpose of the modelling approach in this study is to evaluate the effectiveness of the actions recommended to help maintain resource availability and prevent excess deaths and to provide policy recommendations based on the modeling results. We do this by estimating the number of deaths prevented by applying a combination of several actions.

In this study we wish to answer the questions listed below.

- What role have the public and private healthcare systems been playing vis-a-vis the provision of health care resources during the pandemic in Gauteng province, South Africa?
- What management strategies have been implemented to optimize the limited healthcare re- sources and how effective have they been?
- What scenarios could result in the eventuality of excess deaths due to scarcity of healthcare resources? what would be the predicted number of deaths?
- How long before the province faces resource depletion in case of higher number of confirmed COVID-19 cases?

In order to answer these questions, we conducted an observational retrospective study of the anonymized data of COVID-19 patients admitted to hospitals in the province of Gauteng in South Africa. We then developed a SIR-type [25, 26] agent-based model to simulate the in-hospital dynamics of the COVID- 19 patients requiring healthcare resources with several healthcare management strategies in place.

## 2 Materials and methods

### Study setting and data sources

Gauteng is the most populated province of South Africa and has the highest population density in the country (population of Gauteng was 12,914,800 in 2014). According to the 2017 study on the distribution of hospitals and intensive care unit beds in South Africa, Gauteng province has the highest total number of hospitals, total number of hospital beds, total number of surgical beds and the number of hospitals in private sector (0.86, 225.95, 56.44, and 0.66 per 100,1000 people, respectively) [8]. Multiple sources of data were utilized for this study: the hospital data consisting of anonymized patients hospitalized with COVID-19 in Gauteng facilities during the period of March 05, 2020 to October 28, 2021, provided by the Gauteng Department of Health and the number of cases, deaths and recoveries for the same period of time, publicly available at https://www.covid19sa.org/.

### Ethical considerations

The data used for our study was collected by the government of Gauteng province in South Africa (the owner of the data and a co-author in our manuscript). In the context of South Africa, the Government does not obtain ethical approval before collecting data in hospitals. The premier office represented by Mr. Mduduzi Mbada is a co-author in the manuscript and approves the study.

### Observational data analysis

In this study we conducted a retrospective observational study using the data on the COVID-19 patients, admitted to facilities in Gauteng between March 05, 2020, and October 28, 2021. Time series of hospitalizations for different types of beds by date of admission, as well as time series of COVID- 19 deaths and discharge by the outcome date were generated from the data. We calculated outcomes of interest for all facilities and for public and private sector such as in-hospital death rates and pro- portion of patients requiring ventilators. The Statistics and Machine Learning Toolbox in Matlab was used for data pre-processing and data analysis. Several distributions were used to fit the length of stay (LOS) for hospitalized patients and the Landau distribution was selected as the best fit. Landau distribution is a fat-tailed distributions where the distribution is largely skewed compared to a normal or exponential distribution, with parameters *c* for scale and *μ* for location. The distribution fitting was done for patients hospitalized in all types of beds, general and Isolation ward, and high care and ICU beds, during the first, second and third wave. The LOS were also fitted for public and private sector, separately for each wave. The average length of stay (ALOS) the for the ward and ICU beds were estimated from the data, during each wave. For the LOS fitting we included patients who have been discharged, transferred, or have died during the study period, and excluded those who were still in hospital. We also removed negative LOS values and excluded the outliers by considering the 99% percentile range for each wave. The distribution fitting was completed using the Mathematica software, version 11.1.1.

### Modelling approach

The dynamics of the disease is captured by an agent-based model while keeping track of the available healthcare resources and the possibility of increasing the resources depending on the demand.

**Fig 1:**
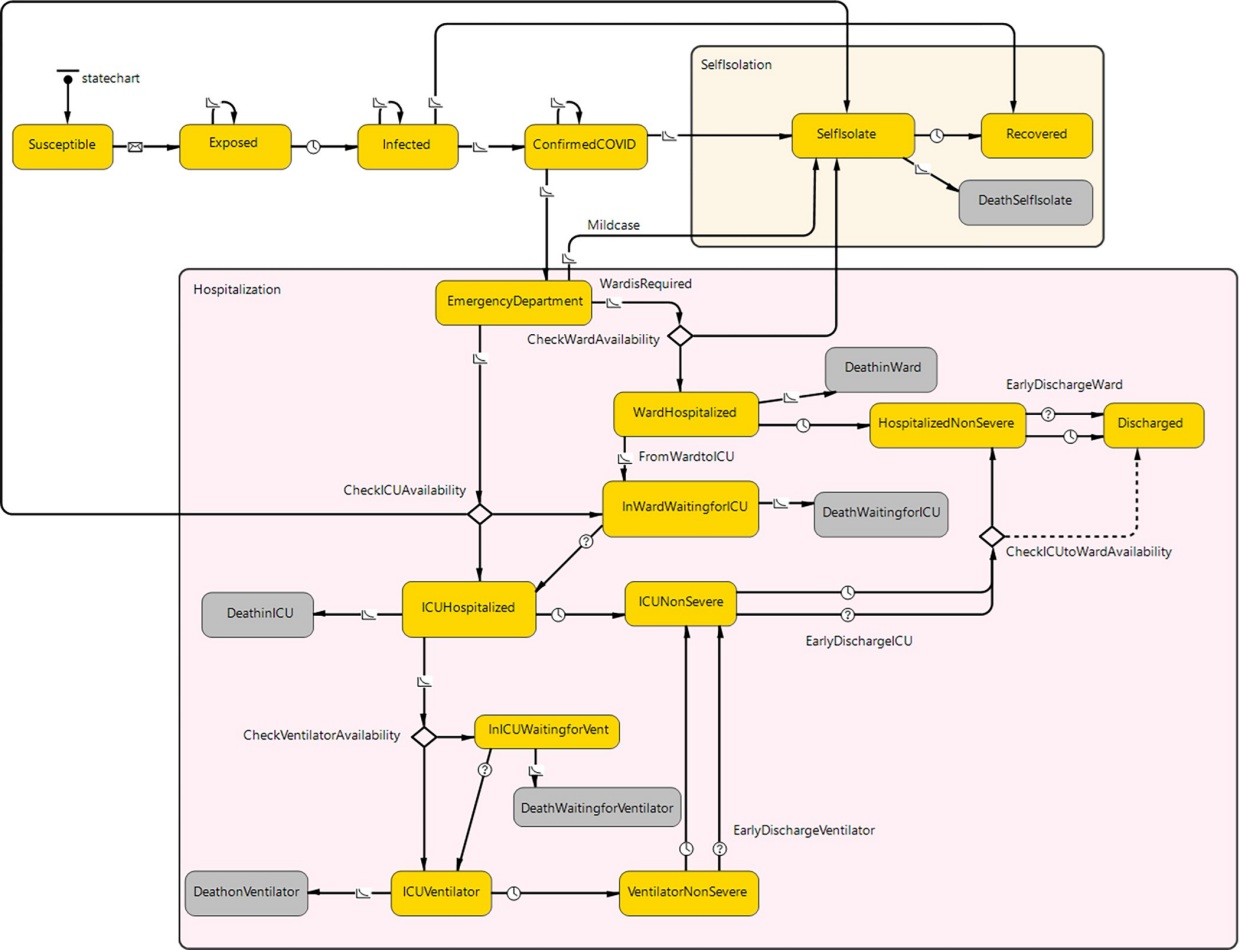
Flow diagram of the disease dynamics and the in-hospital flow of COVID-19 patient in AnyLogic.

This is implemented in AnyLogic (version 8.7.6 University). The susceptible individuals move to the exposed state as a result of a contact with an exposed, infected or confirmed individual. The rate of transmission is different if the contact is between an exposed, infected (but not confirmed) or con- firmed individual. Exposed individuals move to infected state at a given rate and a fraction of infected individuals get confirmed. Given a wide bound of asymptomatic fraction for COVID-19 provided by several studies and the uncertainty on transmission of COVID-19 from asymptomatic cases [27, 28], we excluded the asymptomatic infections as well as the infections with mild symptoms due to limited testing of mild cases. Therefore, the disease confirmation rate refers only to symptomatic cases with severe symptoms. Infected individuals who are not confirmed recover at a given rate. A fraction of confirmed individuals self-isolate and therefore will not transmit the disease anymore. They may recover or die (with a very low possibility). Confirmed individuals with severe symptoms visit the emergency department (ED). There are three possibilities: they may be admitted to the hospital with either an ICU bed or a ward bed; if hospitalization is not required then they will be sent home for self-isolation. We assume that individuals who self-isolate will not visit ED for a second time. Avail ability of the ICU and ward bed is checked, and decisions are made based on that. For instance, if an ICU bed is needed, then we check for ICU availability: if available the patient will be hospitalized in an ICU bed if not, he/she will be admitted to a ward bed and wait for an ICU bed. If none of these are available, then the patient will be required to self-isolate. ICU patients who need ventilators are moved to a ventilator unit, if available, otherwise they wait for a ventilator. Previously ward hospital ized patients may need an ICU bed and will move to an ICU bed if available. State of the ward and ICU bed hospitalized patients is divided into severe and non-severe and patients in non-severe states can be discharged earlier than the baseline duration of hospitalization to free beds when the capacity is limited. All ICU hospitalized patients move to a ward bed before being discharged, unless the number of available ward beds is low and, in that case, they will be discharged directly from ICU. We assume patients in an ICU bed with ventilation move to an ICU bed if they do not need ventilation anymore. Patients on ventilator also have two states of severe and non-severe and they can move to an ICU bed without ventilator depending on the number of new patients in critical need for ventilation. A fixed average length of stay (ALOS) is considered for three types of resource use (ward bed, ICU and ICU with ventilation). All patients will eventually move to the non-severe state of ward hospitalization, and they will be discharged. There is a possibility of deaths for some of the states in the hospital: patients may die during a severe stage of ward bed hospitalization and severe stage of ICU and during ventilation (resource-independent probability of death while receiving the required service). Patients waiting for an ICU bed, or a ventilator die with a high probability (resource-dependent probability of death). A flow diagram of the agent-based model is given is Fig 1.

In order to simulate a realistic plot, we utilized the observed data from Gauteng province in South Africa. To evaluate the effectiveness of the strategies used for the healthcare resources management we took the following steps. We used eight data sets, i.e., time series of the daily number of hospital admissions, general ward, ICU admissions, number of patients on a ventilator and the cumulative number of recoveries, discharged and in-hospital deaths reported by Gauteng hospitals during three COVID-19 waves, to estimate the model parameters. The model calibration was performed using an Anylogic built in optimization method with the following model variables: the daily number of new confirmed cases, daily number of new ward/isolation and HC/ICU admissions, daily number of new patients requiring ventilation, cumulative number of discharged patients, cumulative number of in-hospital deaths and the cumulative number of recoveries. We considered nine different scenarios: the three epidemic waves in Gauteng represented a low, moderate and high epidemic and three levels of resource capacity for each of these epidemic waves. Parameter values were estimated and fixed for each epidemic wave. Finally, we applied a combination of different resource management strategies and calculated the number of days to resource depletion and the number of excess deaths (as a result of resource scarcity) for each of these scenarios.

It is important to note that an accurate estimate of the total number of general ward, isolation ward, high care and ICU beds, as well as the number of ventilators in the province were not available and therefore an interval for the maximum number of available resources was considered. While the province of Gauteng has a higher number of ward and surgical hospital beds compared to other provinces [8], we set the intervals to be lower than these numbers to demonstrate the importance of resource management strategies in situations where the available resources are low. We considered the following intervals for each of these resources: [7000, 10000] for general and isolation ward beds, [2000, 4000] beds for HC/ICU beds and [800, 1200] for the number of ventilators available.

Model parameters. The parameters used in this study were collected from different resources, separately for each epidemic wave in Gauteng: a) estimated from the hospital data; b) data-fitting using the model; c) from literature. The list of parameters and their values are given in Table B in S1 Text.

Pandemic scenarios and management strategies. A number of scenarios are considered based on different factors affecting the availability of resources, magnitude of the COVID-19 epidemic and the severity of symptoms (e.g., different variants with higher infectivity, longer duration of illness and more severe). Here we consider the following: (i) three cases for the disease dynamics: high, moderate and low number of confirmed COVID cases from the first, second and third wave in Gauteng, (ii) three cases for the resource capacity: large, medium and low capacity for the maximum number of resources. Since we do not have an accurate estimate of the number of resources, we consider an upper bound for the maximum number of resources. Two general resource management strategies are considered:

A) Increasing the resources when the bed occupancy is high by adding 25% of the maximum capacity. This could be done via several actions such as allocating more hospitals to COVID patients, opening closed units, converting single rooms to double rooms if possible and rescheduling or cancelling the non-surgery procedures and elective surgeries.

B) early discharge of COVID patients when they are in the improved stage for both ward and ICU beds as the number of available resources drop below a certain threshold. This strategy consists of three components: identifying ward bed hospitalized patients in the improved stage and discharging them when the ward beds available are low; identifying ICU hospitalized patients in the improved stage and moving them to a ward bed when the number of ICU beds are low and there are enough available ward beds; discharging ICU patients directly from ICU when ward beds are low.

In all of these scenarios, we started by allocating 50% of the maximum available resources to COVID- 19 patients. A combination of these strategies were implemented in the agent-based model to estimate the time to resource depletion as well as the number of excess deaths due to shortage of resources for the above scenarios. In other words, we have estimated the number of resource-dependent deaths that have been averted by implementing various strategies.

## 3 Results

In this section we present the results from the two methods that we have used in the study: the observational data analysis and the agent-based modeling.

### Observational analysis results

During the study period (March 05, 2020, to October 28, 2021) a total of 125, 751 patients were admitted to 134 facilities in Gauteng. The study period includes three waves of the pandemic: the first wave is between March 05 to November 15, 2020, the second wave between November 16, 2020, to April 15, 2021, and the third wave between April 16 to October 28, 2021. We observed differences between the third wave and the first two waves. A total of 33, 689, 32, 558 and 59, 496 patients were admitted to the hospital during these three waves, respectively. The percentage of patients admitted to a high care (HC) or an ICU bed were 15.96%, 16.35% and 12.73% and the percentage of patients admitted to a HC/ICU bed who were ventilated, were 40.93%, 53.96% and 68.6% (these numbers were calculated as the total number of patients who were ventilated during their hospital stay to the total number of patients admitted to a HC/ICU bed, based on the assumption that only HC and ICU beds were equipped with ventilators). The length of stay (LOS), in days, was also different for the three waves. The 99% percentile ranges were [1, 118], [1, 76], [1, 58], which means that 99% of patients stayed in hospital up to 118 days during the first wave, up to 76 days in the second wave and up to 58 days during the third wave.

The total in-hospital deaths for all types of bed during the three waves were 18.74%, 22.06% and 23.66%. The percentage of patients who have been ventilated at least once during their hospital stay and died from COVID-19 were 61.24%, 59.24% and 61.32%. The beginning and end time of ventilation was not provided so it is not known whether a patient died on a ventilator. The state at which a patient had died was not available. Additional information on the patients’ characteristics such as mean age, male/female and underlying conditions is provided in Table 1.

**Table 1:** Patient characteristics.

Data period	Bed type	Mean age (Standard deviation)	Female (%)	One or more underlying conditions (%)
First wave	All	53.1 (21)	54.4	28.9
	HC/ICU	55.3 (17.5)	45.7	46
	On ventilator	57.7 (16.8)	43.8	47.4
Second wave	All	51.9 (18.7)	53.5	25.8
	HC/ICU	55.9 (17)	48.2	27.1
	On ventilator	56.1 (14.9)	45.6	37.3
Third wave	All	53.2 (19.7)	51.5	25
	HC/ICU	55.1 (18.9)	44.3	28.1
	On ventilator	56.5 (16.2)	43.6	32.9

A normal distribution was fitted to the age of hospitalized patients for all types of beds as well as those admitted to a high care or ICU bed and those on a ventilator. More than half of the patients admitted to hospital were female for all three waves while the percentage of female patients in high care or ICU and on a ventilator were less than male patients. The proportion of patients with at least one underlying condition among those admitted to a high care or ICU bed and on a ventilator were high.

**Fig 2:**
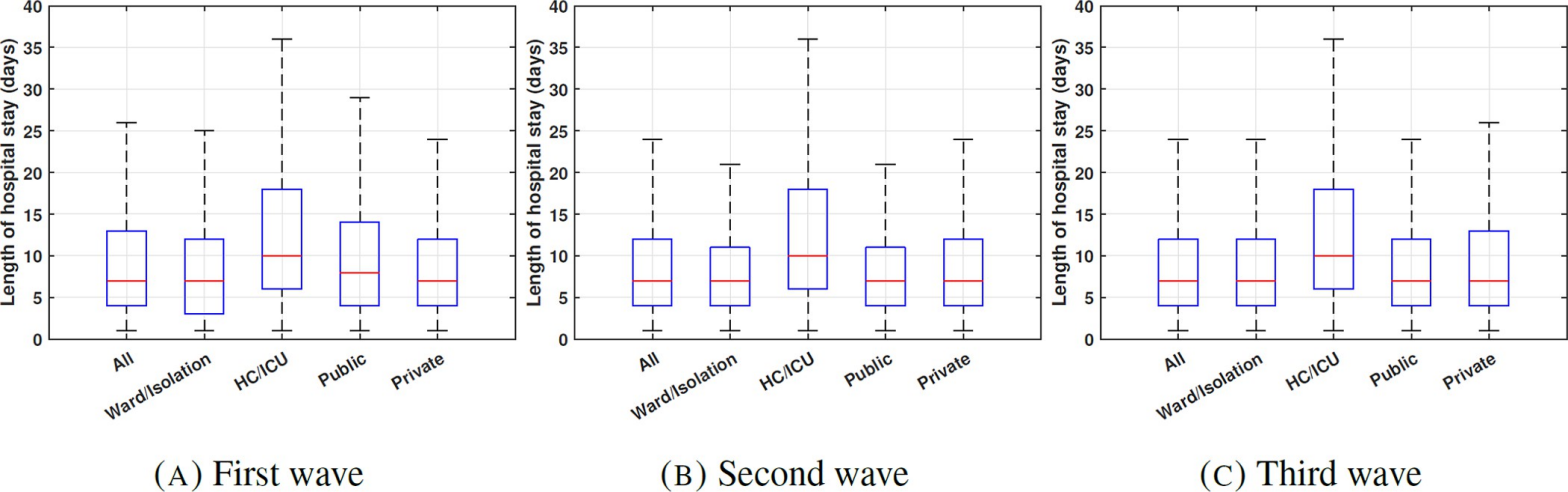
Distribution of the length of hospital stay during the first, second, and third wave of the pandemic.

**Fig 3:**
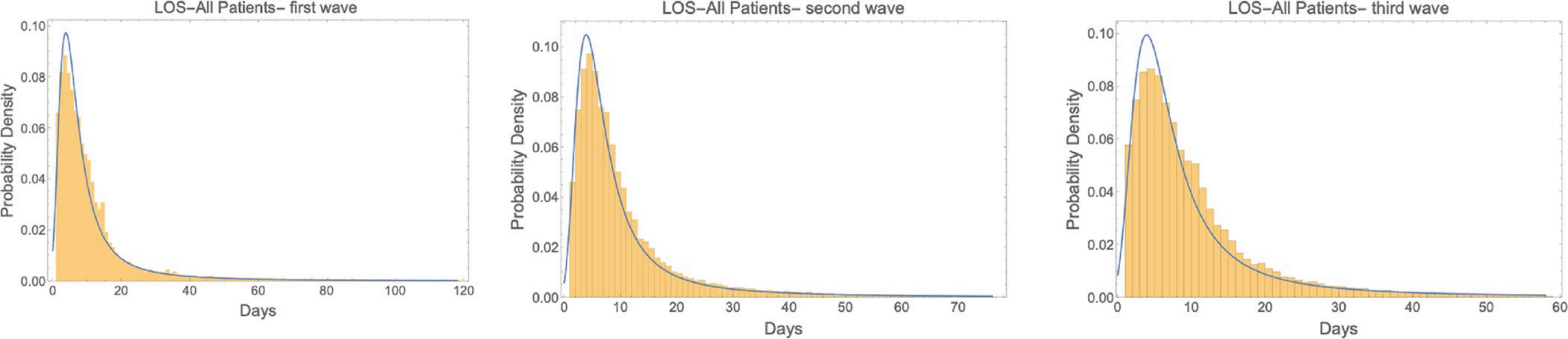
LOS for all hospitalizations in three waves in Gauteng, SA.

LOS distribution fitting. The distribution of the hospital stays (box plots) for all hospitalizations, general/isolation ward, HC/ICU, public and private sectors for the three waves are given in Fig 2. The length of stay for patients in all facilities, as well as public and private facilities were fitted to a Landau distribution. Only the positive entries in the 99% percentile range were included in the distribution fitting. Based on the fitted distribution, the median of hospital stays for HC/ICU beds are higher than general/isolation ward beds for all three waves (9.97 and 6.22 for the first wave, 10.05 and 6.17 for the second wave, and 10.21 and 6.23 for the third wave). The median of all hospital stays is about the same in public and private sectors (7.01 and 6.54 for the first wave, 6.79 and 6.63 for the second wave, and 6.67 and 6.83 for the third wave). All results are summarized in Table A in S1 Text. Fig 3 shows the fitting plots for all patients during the three waves. The fitting plots for public and private sectors as well as Ward and ICU hospitalizations are given in the Figs A–F in S1 Text.

Proportion of hospitalizations with long hospital stays were calculated separately for the three waves and in five categories: all hospitalizations, general/isolation ward hospitalizations, HC/ICU hospitalizations in both sectors and all hospitalizations in public and private sector. The proportions are calculated for length of hospital stays more than 10, 20, 30, 40 and 50 days. Results are given in Table 2.

**Table 2:** Proportion of long hospital stays.

Data period	Bed type/sector	LOS > 10 days	LOS > 20 days	LOS > 30 days	LOS > 40 days	LOS > 50 days
First wave	All	0.33	0.12	0.07	0.04	0.03
	Ward/Isolation	0.3	0.11	0.07	0.04	0.03
	HC/ICU	0.48	0.21	0.1	0.06	0.03
	Public	0.35	0.13	0.09	0.07	0.06
	Private	0.31	0.12	0.06	0.03	0.01
Second wave	All	0.29	0.09	0.04	0.02	0.01
	Ward/Isolation	0.25	0.07	0.03	0.01	< 0.01
	HC/ICU	0.49	0.2	0.1	0.05	0.03
	Public	0.29	0.08	0.04	0.02	< 0.01
	Private	0.3	0.11	0.05	0.02	< 0.01
Third wave	All	0.31	0.1	0.04	0.01	< 0.01
	Ward/Isolation	0.29	0.08	0.03	0.01	< 0.01
	HC/ICU	0.49	0.2	0.09	0.04	0.02
	Public	0.31	0.08	0.03	0.01	< 0.01
	Private	0.32	0.11	0.04	0.02	< 0.01

Probability of LOS more than 10, 20, 30, 40 and 50 days are calculated for each wave in five categories: all hospitalizations, general/isolation ward hospitalizations, HC/ICU hospitalizations in both sectors and all hospitalizations in public and private sector.

We observe that a higher proportion of all hospitalizations have a LOS of more than 30 days during the first wave (0.07) compared to the second and third waves (0.04). We note that a higher proportion of patients hospitalized in a high care or ICU bed have a hospital stay longer than 20 days compared to those hospitalized on an isolation or general ward bed during the three waves (about 10% for ward beds and about 20% for HC/ICU beds). Also, during the first pandemic wave, more hospitalizations have LOS longer than 40 and 50 days in public facilities compared to private facilities (the proportion of hospitalizations with LOS longer than 50 days are 0.06 and 0.01 in the public and private sectors, respectively).

### Public and private sector

**Fig 4:**
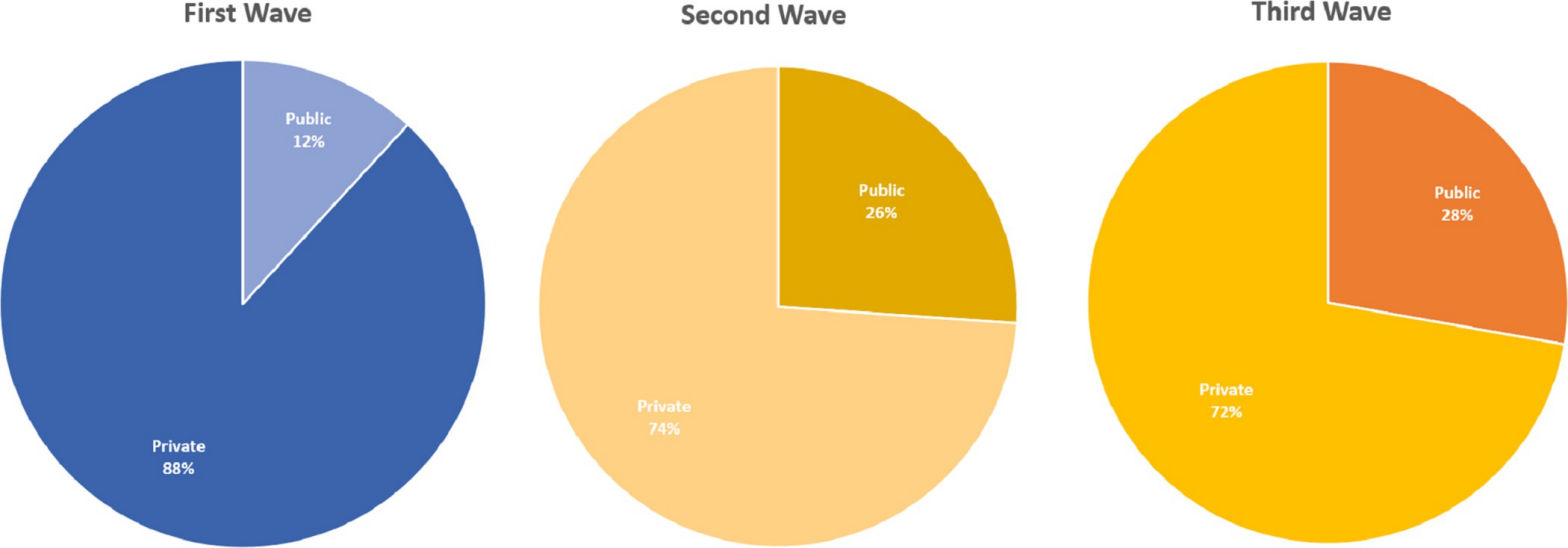
Proportion of patients hospitalized to HC/ICU beds in public and private sectors during the three waves.

COVID-19 patients were hospitalized in 40 public and 94 private facilities. The total number of patients in public and private facilities were 53, 228 and 72, 523, respectively. The differences between public and private sector were also notable during the three waves, given that approximately 75% of the ICU beds are available at private facilities while about 80% of the South African population rely on the public sector. During the first wave, 11.86% of all HC/ICU patients were in public facilities while 88.41% were in private facilities. The numbers for the second wave are 26.09% and 74.04% and in the third wave 27.77% and 72.23% (see Fig 4). The in-hospital deaths in public hospitals were higher than private hospitals during all three waves. The results are given in Table 3.

**Table 3:** In-hospital deaths in public and private facilities.

Epidemic wave	Sector	In-hospital deaths (%)	Ward/Isolation deaths (%)	HC/ICU deaths (%)
First wave	All Facilities	18.8	16.1	32.7
	Public	24	22.9	49.8
	Private	14.5	9	30.5
Second wave	All Facilities	22.1	18.8	38.8
	Public	27.1	26.2	35.8
	Private	17.9	11.8	39.9
Third wave	All Facilities	23.7	21.5	38.4
	Public	26.8	26.5	30.4
	Private	21.6	18	41.5

**Fig 5:**
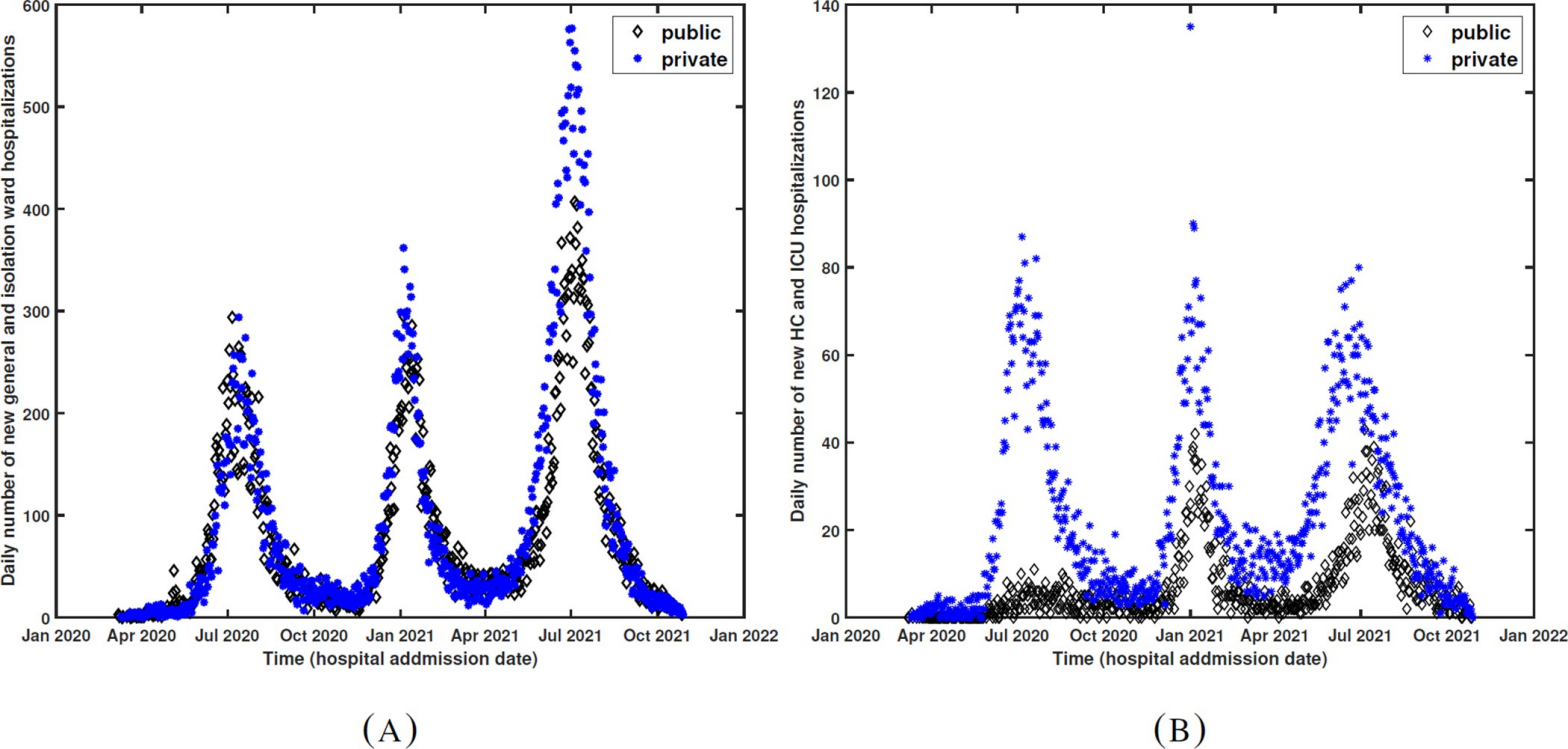
(A) Daily number of general/isolation ward hospitalizations in public (black diamonds) and private (blue stars) sectors (B) Daily number of HC/ICU hospitalizations in public (black diamonds) and private (blue stars) sectors.

We can see that the death rates among ward bed hospitalized patients are significantly higher in public sector during all three waves, however, the deaths rates among HC and ICU bed patients are higher in private facilities during the second and third waves. Fig 5 shows time series of the daily number of ward/isolation and HC/ICU hospitalizations in public and private sectors.

**Fig 6:**
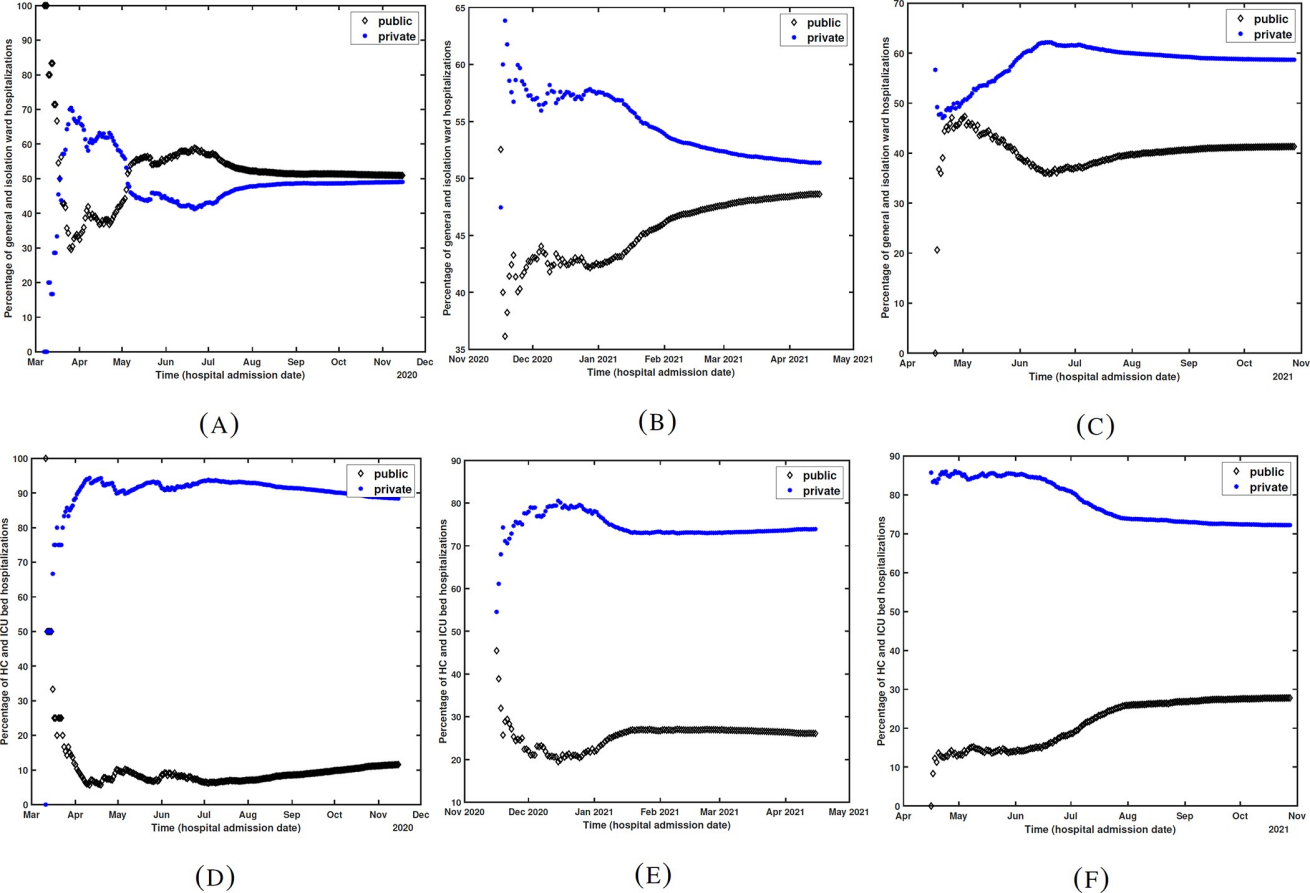
(A-C) Percentage of general/isolation ward hospitalizations in public and private sectors over time during the three waves, respectively (D-F) Percentage of HC/ICU hospitalizations in public and private sectors over time during the three waves, respectively (100*×*cumulative number of ward (ICU) hospitalizations in public (private)/cumulative number of ward (ICU) hospitalizations in both sectors).

During the first wave the number of ICU hospitalizations were significantly higher in private sector than in the public sector. During the second and third waves, the number of ICU hospitalizations in the public sector were higher than the first wave (particularly during the peak time). This shows that more ICU beds were allocated to COVID-19 patients in public sector (Fig 5B). The time series of ward and isolation hospitalizations are about the same for public and private sectors during the first two waves and slightly higher in private sector, during the peak time of the third wave. This shows that more general ward beds were made available in the private sector for the third wave (Fig 5A). This indicates that both public and private sectors were engaged to provide the necessary care to COVID patients. Fig 6 shows the percentage of cumulative ward/isolation and HC/ICU bed hospitalizations, separately for the three waves, in the public and private sectors.

### Modeling results

We implemented a combination of management strategies in our model. Many combinations of the actions and variations of their timing and criteria are possible, however, we selected a few to demonstrate how the model can help us evaluate the effectiveness of these actions. The results of the simulations with the agent-based model are summarized in Table 4.

**Table 4:** Scenario analysis results.

Epidemic Size and Maximum Resource Capacity*	Management Strategies	Number of deaths averted by this strategy
High-Low	A only (versus no strategy)	1016
	A and B-case 1 (versus A only)	297
	A and B-case 2 (versus B-case 1)	158
High-Medium	A only (versus no strategy)	1278
	A and B-case 1 (versus A only)	195
	A and B-case 2 (versus B-case 1)	1
High-High	A only (versus no strategy)	957
Medium-Low	A only (versus no strategy)	1067
	A and B-case 1 (versus A only)	297
	A and B-case 2 (versus B-case 1)	5
Medium-Medium	A only (versus no strategy)	1003
	A and B-case 1 (versus A only)	12
Medium-High	A only (versus no strategy)	633
Low-Low	A only (versus no strategy)	1366
	A and B-case 1 (versus A only)	22
Low-Medium	A only (versus no strategy)	408
Low-High	A only (versus no strategy)	157

Number of deaths averted by implementing a combination of strategies for each epidemic scenario. The scenarios are based on the epidemic size (high, medium and low) and the maximum capacity of the resources (high, medium and low). for each scenario we have three management strategies based on different actions. The number of deaths prevented by the strategy implemented measures how effective that strategy is. It is clear that by adding more timely actions and changing the criteria a high number of deaths can be prevented. Action A is increasing the available resources by 25–35%. Action B-case 1 allows early discharge of ward, ICU and ICU patients on ventilator when the bed/ventilator capacity is below 5% of the maximum capacity and when there is at least 1 ward bed available. Action B-case 2 allows early discharge of ward, ICU and ICU patients on ventilator when the bed/ventilator capacity is below 10% of the maximum capacity and when there is at least 10% ward bed available.

* The maximum resource capacity refers to the total number of hospital beds (ward and ICU) and number of ventilators in the province. Initially 50% of this capacity is allocated to COVID-19 patients.

The first column of the table represents the epidemic waves in Gauteng and the maximum resource capacity. The first wave is considered as a low epidemic curve, the second is a medium curve and the third wave is a high epidemic curve. The three values for maximum resource capacity are marked as low, medium and high. The second column shows which strategies have been used, and the timing/criteria for the actions. The last column shows the number of deaths averted by taking these actions. The timing of taking actions and the criteria for the actions are critical. In our model, we keep track of available general/isolation ward beds, HC/ICU beds and ventilators. As the number of hospitalizations increases, we allow for an increase of the number of resources (as a result of conducts such as allocating more hospitals to COVID patients, canceling or rescheduling elective surgeries or non-surgery procedures). We also allow for early discharge of patients based on the improvement of their health condition. Whether ICU patients can be moved to a ward bed before being discharged also depends on the number of available ward beds. For example, if less than 400 beds are available, we start discharging ward hospitalized patients. If less than 150 ICU beds and more than 400 ward beds are available, we move ICU patients to a ward bed, otherwise (i.e., ward beds are less than 400) we discharge them directly from ICU. The threshold values of available beds were varied, and we see that a delay in taking these actions result in early resource depletion with a higher number of resource-dependent deaths.

For each scenario of epidemic size with a given resource capacity, first we ran the model without any actions and then we added strategy A and estimated the number of deaths prevented by applying strategy A. Then we added strategy B with two cases: in case 1 we discharge ICU patients when the ICU bed capacity is less than 5% of maximum bed capacity and move them to a ward bed given at least 1 bed is available; in case 2 we discharge ICU patients when the ICU bed capacity is less than 10% of maximum bed capacity and move them to a ward bed given at least 5% of maximum ward bed capacity is available. Then we estimated the number of deaths prevented by applying case 2 instead of case 1. As shown in Table 4, we see that with a high epidemic size and a low resource capacity, adding a 25–35% of the maximum available resources prevents 1016 deaths and early discharge of patients with basic discharge criteria prevents another 297 deaths and finally the early discharge of patients with a better discharge criterion prevents an additional 158 deaths. By taking these actions as the epidemic grows and the number of emergency department visits increases, we can prevent a total of 1471 deaths.

**Fig 7:**
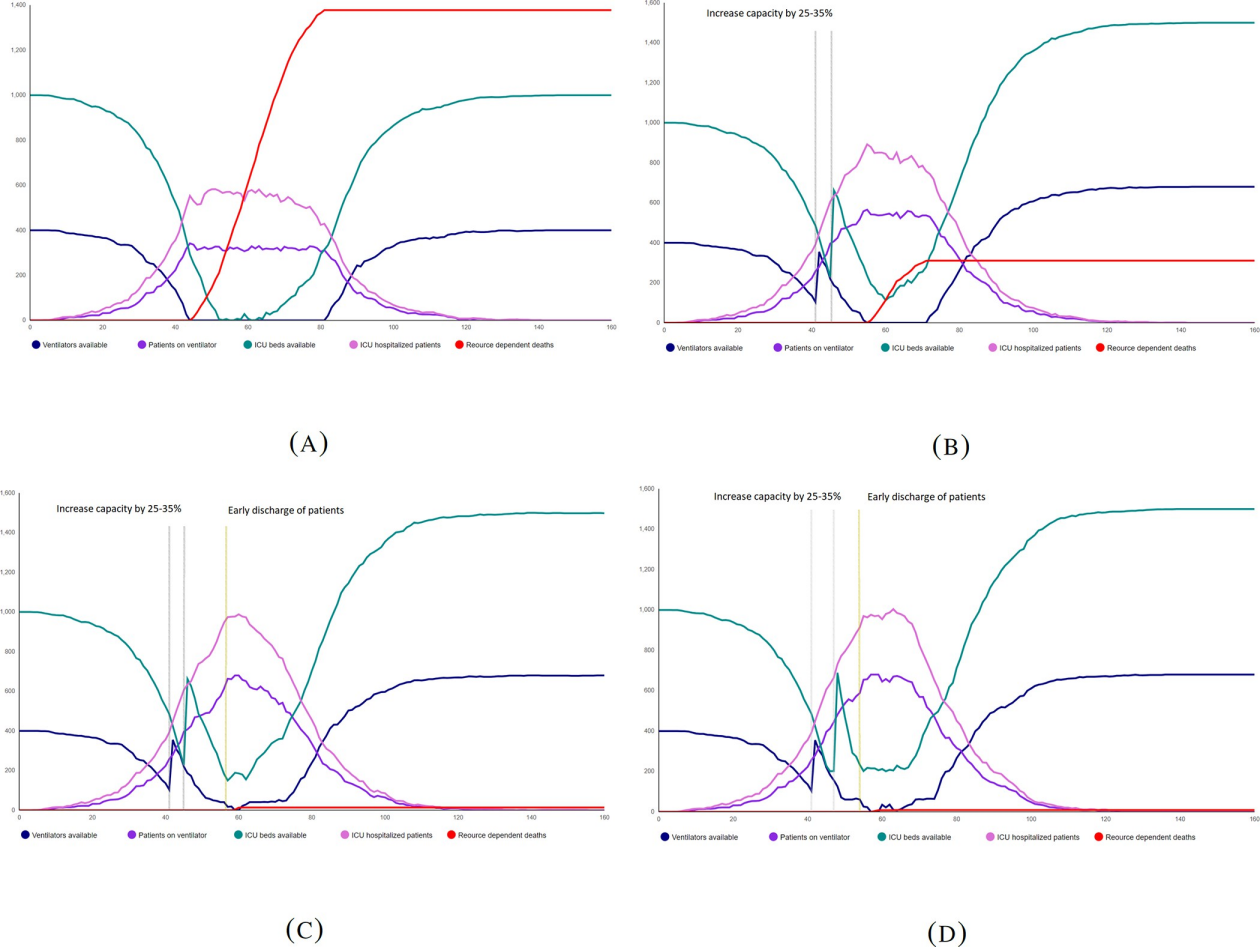
Dynamics of the available resources for HC/ICU beds, ventilators, and the number of resource dependent deaths (A) no management strategy is implemented and therefore the resources are depleted quickly resulting in a high number of deaths (B) 25–35% is added to the resources which reduces the number of resource-dependent deaths significantly (C-D) In addition to the increase of bed and ventilator capacity, early discharge of patients is implemented with two different criteria which further reduces the number of resource-dependent deaths. In (D) the early discharge of ICU patients and patients on ventilator starts when a higher number of resources are still available.

It is clear from these results that if a higher maximum capacity is available, the number of total deaths in the absence of any strategy is lower and that fewer interventions are needed to maintain bed availability. For instance, with the moderate epidemic size the lower bound of resource capacity (7000 ward beds, 2000 ICU beds and 800 ventilators) results in a total of 1378 deaths without any strategies implemented while the upper bound of the resource capacity (10000 ward beds, 4000 ICU beds and 1200 ventilators) results in a total of 633 deaths when no management action is in place. Therefore, the management strategies are much more crucial when the maximum capacity is low for the country/province. The plots in Fig 7 depict the dynamics of ICU beds, ventilators, patients currently in ICU, patients currently on a ventilator, as well as the number of resource-dependent deaths, for a moderate epidemic size and a low resource capacity.

These plots show how the number of resource-dependent deaths decreases by implementing management policies and by taking timely actions.

This model can also predict the time to resource depletion which can help us take timely actions. These results are summarized in Table 5. It is clear that by implementing each of these strategies we can delay the time to resource depletion and with some strategies the resources will not be depleted. For instance, with a medium epidemic size and a low resource capacity, the resources will be depleted after 44 days if no management strategy is in place, while with the increase of resources and timely discharge of patients, the resources will not be depleted. It is important to note that when the epidemic size is high and the maximum resource capacity is low, the resources will eventually be depleted (High-Low case in Table 5), which highlights the importance of reducing the number of infections in the community to avoid overwhelming the healthcare system. A detailed description of the model and the flow diagrams of the infection-self isolation-hospitalization process (Figs G–K in S1 Text) are available in S1 Text.

**Table 5:** Scenario analysis results for the number of days to resource depletion.

Epidemic Size and Maximum Resource Capacity	Management Strategies	Number of days to resource depletion
High-Low	No strategy	59
	A only	67
	A and B-case 1	70
	A and B-case 2	70
High-Medium	No strategy	61
	A only	71
High-High	No strategy	64
Medium-Low	No strategy	44
	A only	55
	A and B-case 1	59
Medium-Medium	No strategy	47
	A only	61
Medium-High	No strategy	51
Low-Low	No strategy	118
	A only	135
Low-Medium	No strategy	122
Low-High	No strategy	130

The number of days to resource depletion for ventilators with and without management strategies. With no strategies in place the resources would be depleted very quickly. For example, with a moderate epidemic size and a low capacity of healthcare resources, the number of ventilators will reach 0 after 44 days if no action is taken, after 55 days if the capacity is increased by 25–35% and after 59 days if the capacity is increased and patients are discharged early, when possible. The number of ventilators will not be depleted with a timely planning for early discharge of patients (Strategy A and B- case 2).

## 4 Discussion

Planning to accommodate large numbers of patients in events such as pandemics is a challenging task for governments and healthcare systems. Creating surge capacity for an increased volume of patients requiring critical care and specific treatments such as heavy ventilation requires quick and effective strategies to be implemented. Detailed guidelines by healthcare experts should be provided to efficiently manage the scarce resources. During each wave of the COVID-19 pandemic health- care systems have developed strategies to optimize the available healthcare resources and provide necessary care to COVID-19 patients. Studies have been conducted in many countries to predict the increased demand for healthcare resources and to determine whether these resources are adequate for providing essential services to COVID-19 patients throughout the pandemic with a focus on the burden of COVID-19 on ward beds, ICU beds and ventilators [11–16].

The importance of this subject and the fact that the COVID-19 pandemic can become difficult to control with the emergence of new variants [3] motivated us to study this issue more rigorously. While studies have been carried out to address the inhomogeneous distribution of hospital resources in South Africa and the consequent challenges and ethical considerations of sharing and prioritizing the limited resources in the country [9, 20, 21], no studies have been conducted to evaluate the management strategies of healthcare resources in South Africa. In this study we have carried out an observational study on COVID-19 hospitalizations in Gauteng, South Africa from March 05, 2020, to October 28, 2021, to understand the healthcare system capacities for accommodating adequate care. Our observational study has shown that more than 80% of patients were admitted to general/isolation ward bed during each wave of the pandemic. During each wave a high percentage of patients, admitted to a HC/ICU bed, required mechanical ventilation, 40.93%, 53.96% and 68.6%, respectively. A higher percentage of patients had a long hospital stay (30 days or more) during the first wave than the second and third waves. Also, HC/ICU hospitalized patients had longer hospital stays than ward bed hospitalized patients. Given that the healthcare system in South Africa is managed by both public and private sectors and based on the fact that more than 70% of high care and intensive care unit beds are available at private facilities, we have observed that the public and private sectors have worked well together to provide the necessary care to all COVID-19 patients. More than 70% of the patients in HC/ICU beds were hospitalized in private facilities and about 50% of ward beds were also provided by private facilities, during the entire study period. Some differences were observed between the public and private sectors. The overall death rates were higher in public sector during all three waves and the death rates among ward patients were particularly higher in public facilities.

We also developed an individual level dynamic model to evaluate the effectiveness of management strategies during three waves of COVID-19 pandemic in minimizing the resource-dependent deaths. The model was developed to assess the effect of several actions, their timing and criteria on maintaining bed availability while providing adequate care to COVID-19 patients. The model simulations for different scenarios, which were designed based on the epidemic size and healthcare resources capacity in the province, showed that implementing management strategies can reduce the number of resource-dependent deaths, significantly. This was particularly observed for scenarios with a high epidemic size and a medium or low maximum resource capacity, as well as a medium epidemic size and a low resource capacity, as shown in Table 4, where between 1300–1500 deaths have been averted by implementing management strategies. More importantly we can see that, in all scenarios simulated in this study, the number of excess deaths due to resource scarcity can be reduced to zero even when the available resources are low. The model simulations also predicted the number of days to resource depletion. For each scenario and with different strategies in place the number days to resource deple tion for ventilators, summarized in Table 5, were calculated. These results show how improving the management strategies can delay and avoid resource depletion.

## 5 Conclusions

Since the first confirmed cases of COVID-19 pandemic, while non-pharmaceutical interventions have helped reduce the number of COVID-19 cases, a high volume of patients have been hospitalized in Gauteng, South Africa. Differences between the three waves of the pandemic have been observed in terms of the length of hospital stays, the need for ventilators and in-hospital death rates. Public and private sectors have managed to provide critical care to COVID-19 patients despite the inhomogeneous distribution of High care and ICU beds in these two sectors. Our modeling study has emphasized the importance of identifying and implementing effective actions to maintain availability of healthcare resources, particularly when a high number of patients require hospital admission, and the healthcare resources are scarce. Based on the simulation results for 9 different scenarios, it is clear that the timely and effective policies can help reduce a significant number of resource-dependent deaths as well as delay and avoid resource depletion.

### Study limitations: Data and methods

The data used in this study has several limitations related to the purposes of the current study. The possible transfer time of patients from a ward bed to a HC/ICU bed and vice versa and the duration of a patient’s ventilation was not provided. The hospital status of deceased patients (i.e., in ward, in HC/ICU or on a ventilator) was not given.

The model developed in this study also has limitations. For a more realistic simulation of hospital management during COVID-19 other healthcare resources could be included such as nurses, physicians, specialists, critical care technician, etc. This model does not take into account the effect of pharmaceutical and non-pharmaceutical effects on the contact rates in the community.

## Supporting information

S1 Text Supplementary information for management of hospital beds and ventilators in the Gauteng province, South Africa, during the COVID-19 pandemic. Contains Figs A-K, Tables A, B and a detailed description of our agent based model.
(DOCX)

S2 Text Inclusivity in global research. (DOCX)
