# Supplementary material for: Management of hospital beds and ventilators in the Gauteng province, South Africa, during the COVID-19 pandemic
Source: PLOS Glob Public Health. 2022 Nov 2;2(11):e0001113. doi: 10.1371/journal.pgph.0001113 (PMC10022393; doi:10.1371/journal.pgph.0001113)
Supplement: S1 Text — Contains Figs A-K, Tables A, B and a detailed description of our agent based model. (DOCX) [file pgph.0001113.s001.docx]

# Supplementary Information

# Management of Hospital Beds and Ventilators in the Gauteng Province, South Africa, During the COVID-19 Pandemic

Mahnaz Alavinejad^1^, Bruce Mellado^2,3,*^, Ali Asgary^4,*^, Mduduzi Mbada^5^, Thuso Mathaha^6^, Benjamin Lieberman^6^, Finn Stevenson^6^, Nidhi Tripathi^6^, Abhaya Kumar Swain^6^, James Orbinski^7,*^, Jianhong Wu^1,*^, and Jude Dzevela Kong^1,*,^*^†^*

1Africa-Canada Artificial Intelligence and Data Innovation Consortium (ACADIC), Laboratory for Industrial and Applied mathematics, York University, Canada 2Africa-Canada Artificial Intelligence and Data Innovation Consortium (ACADIC), School of Physics, Institute for Collider Particle Physics, University of the Witwatersrand, Johannesburg, South Africa

3iThemba LABS, National Research Foundation, South Africa

4Africa-Canada Artificial Intelligence and Data Innovation Consortium (ACADIC), the Advanced Disaster, Emergency and Rapid Response Program, York University, Canada

5Head of Policy at Gauteng Office of the Premier, Johannesburg 2107,

Johannesburg, South Africa

6University of the Witwatersrand, Johannesburg, South Africa

7Africa-Canada Artificial Intelligence and Data Innovation Consortium (ACADIC), the Dahdaleh Institute for Global Health Research, York University, Canada

*These authors have contributed equally to this work and share last authorship

*†*Corresponding Author: [jdk](mailto:jdkong@york.ca)[ong@york.ca](mailto:ong@york.ca)

Fig A in S1 Text: LOS for hospitalizations in (A) public and (B) private sectors during the first wave in Gauteng, SA.


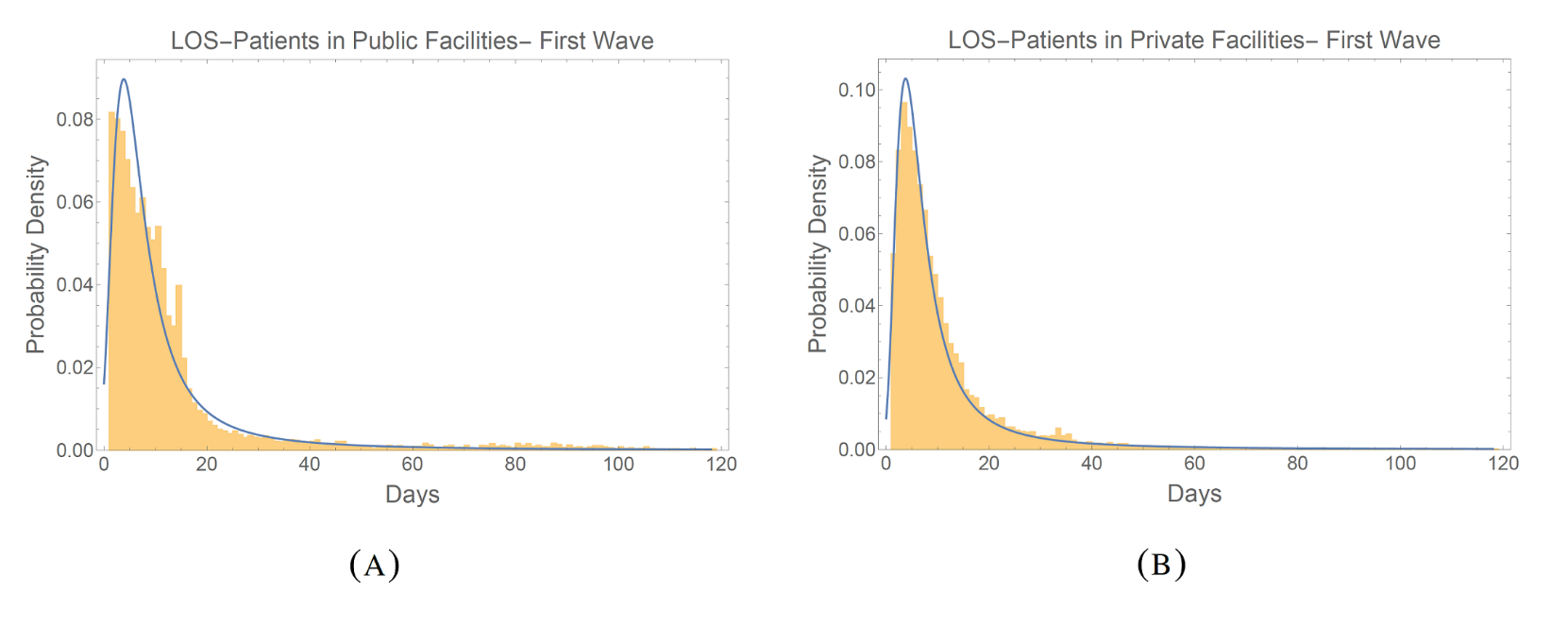


Fig B in S1 Text: LOS for hospitalizations in (A) public and (B) private sectors during the second wave in Gauteng, SA.
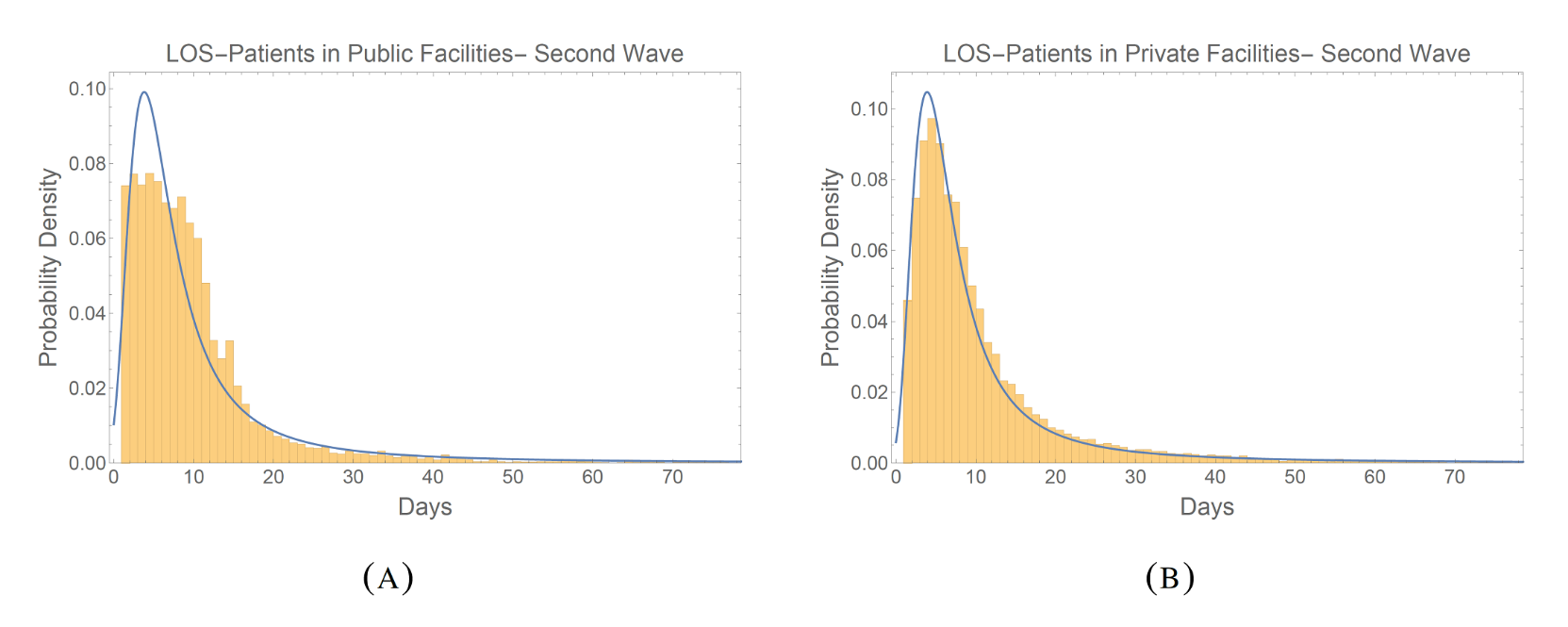


Fig C in S1 Text: LOS for hospitalizations in (A) public and (B) private sectors during the third wave in Gauteng, SA.


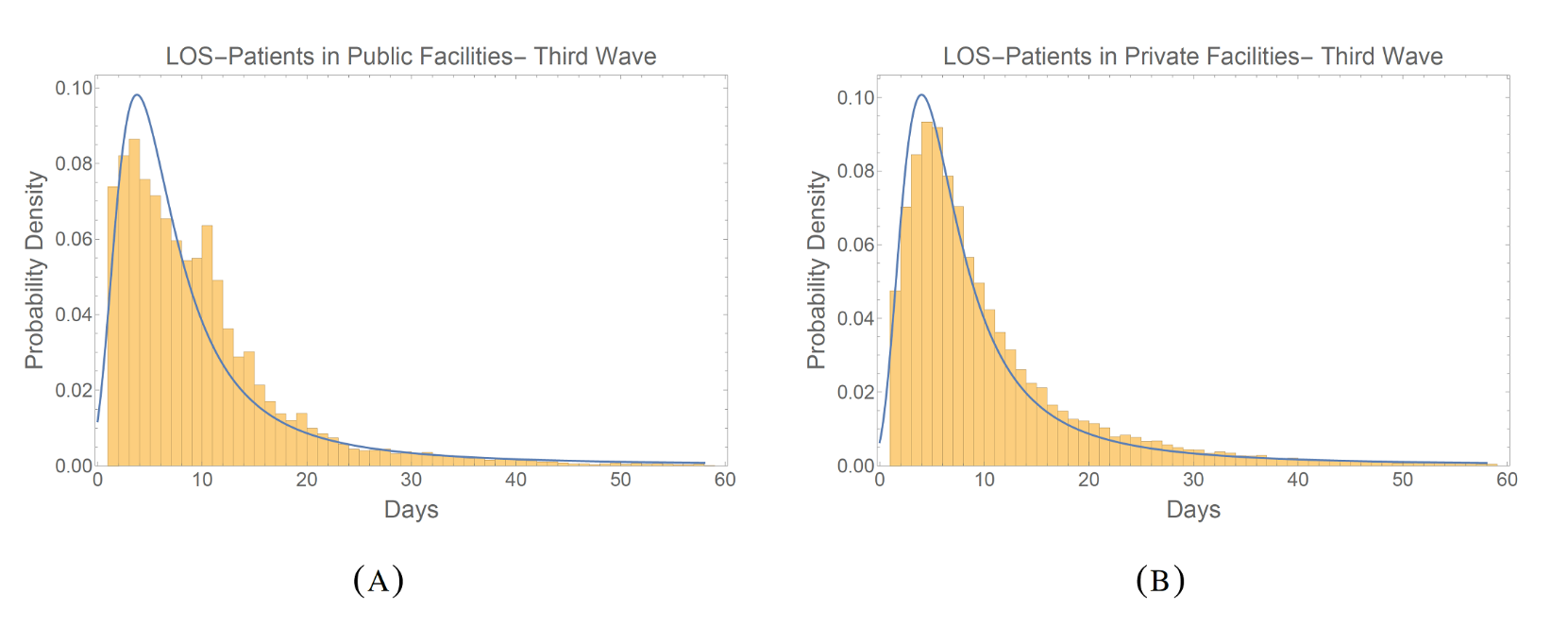


Fig D in S1 Text: LOS for (A) general ward/isolation ward and (B) HC/ICU hospitalizations during the first wave in Gauteng, SA.


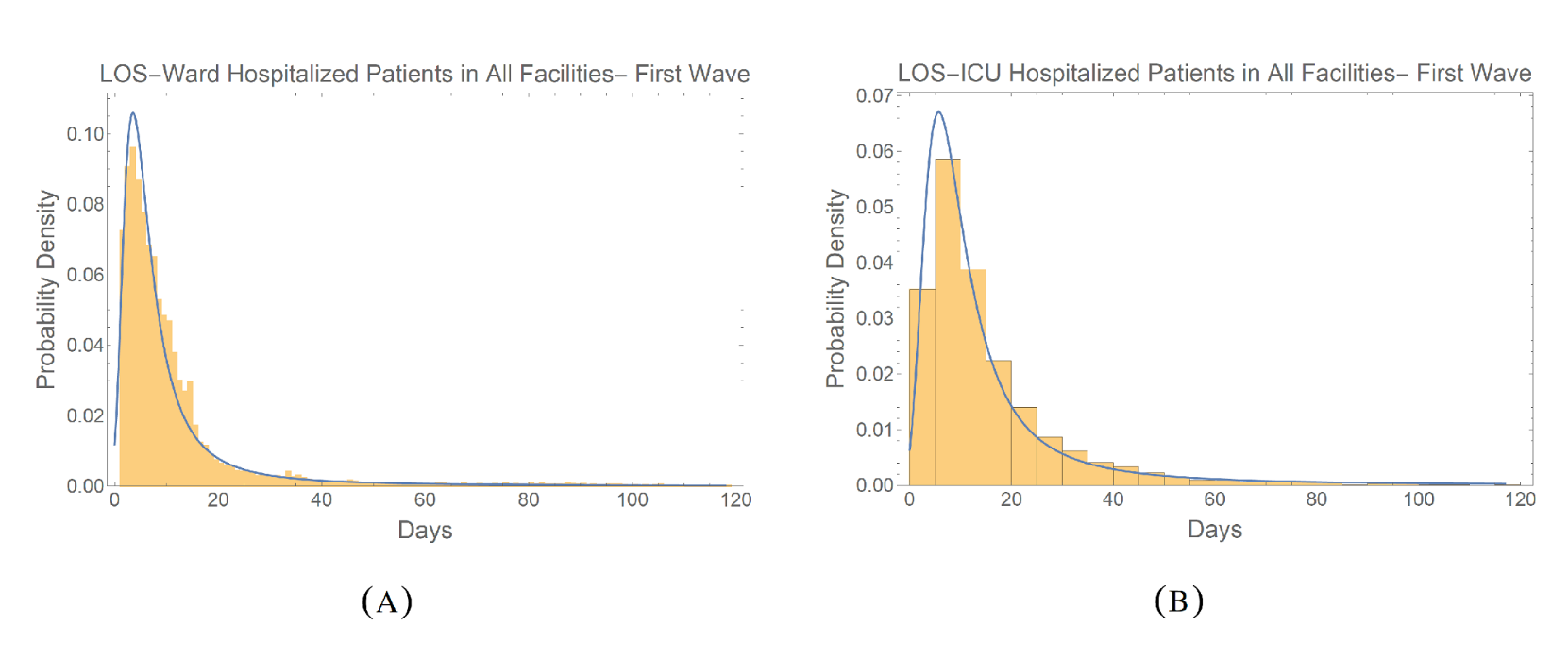


Fig E in S1 Text: LOS for (A) general ward/isolation ward and (B) HC/ICU hospitalizations during the second wave in Gauteng, SA.


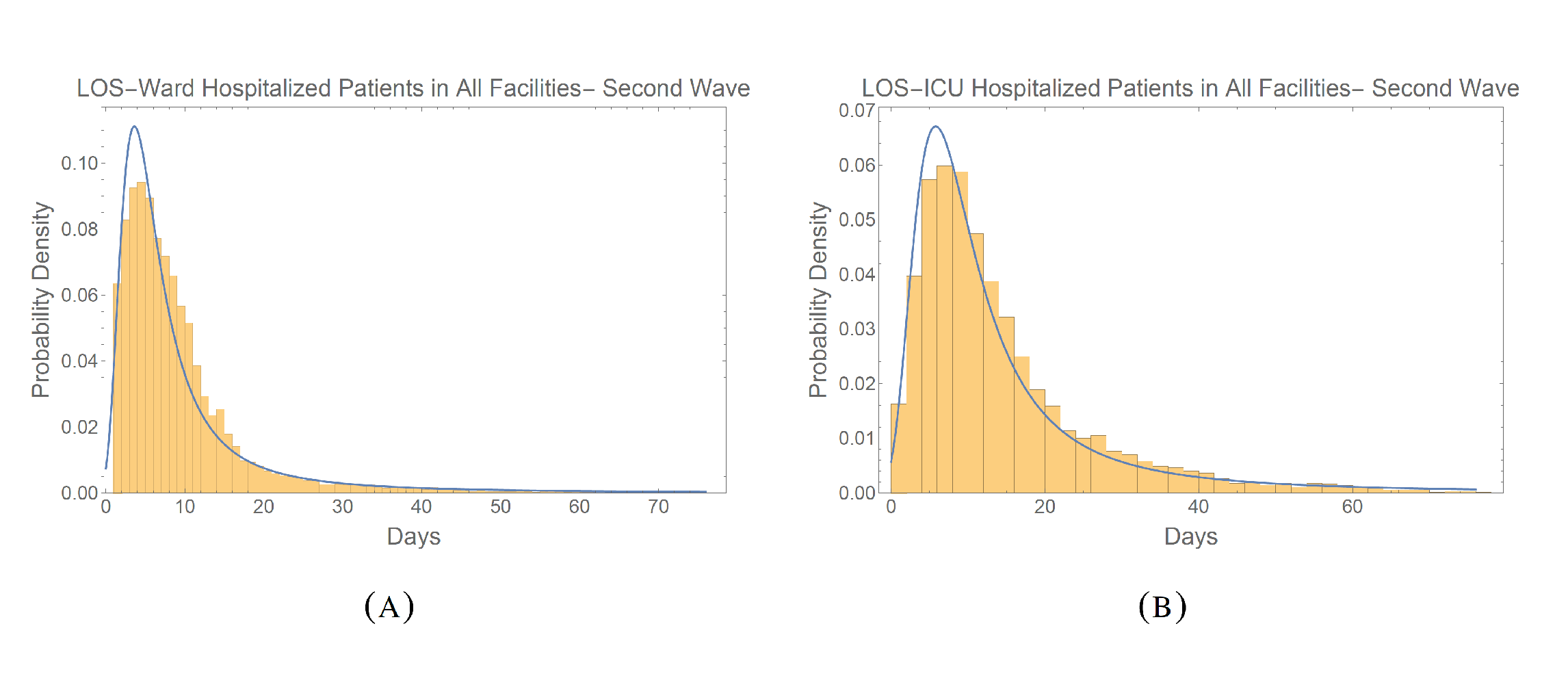


Fig F in S1 Text: LOS for (A) general ward/isolation ward and (B) HC/ICU hospitalizations during the third wave in Gauteng, SA.


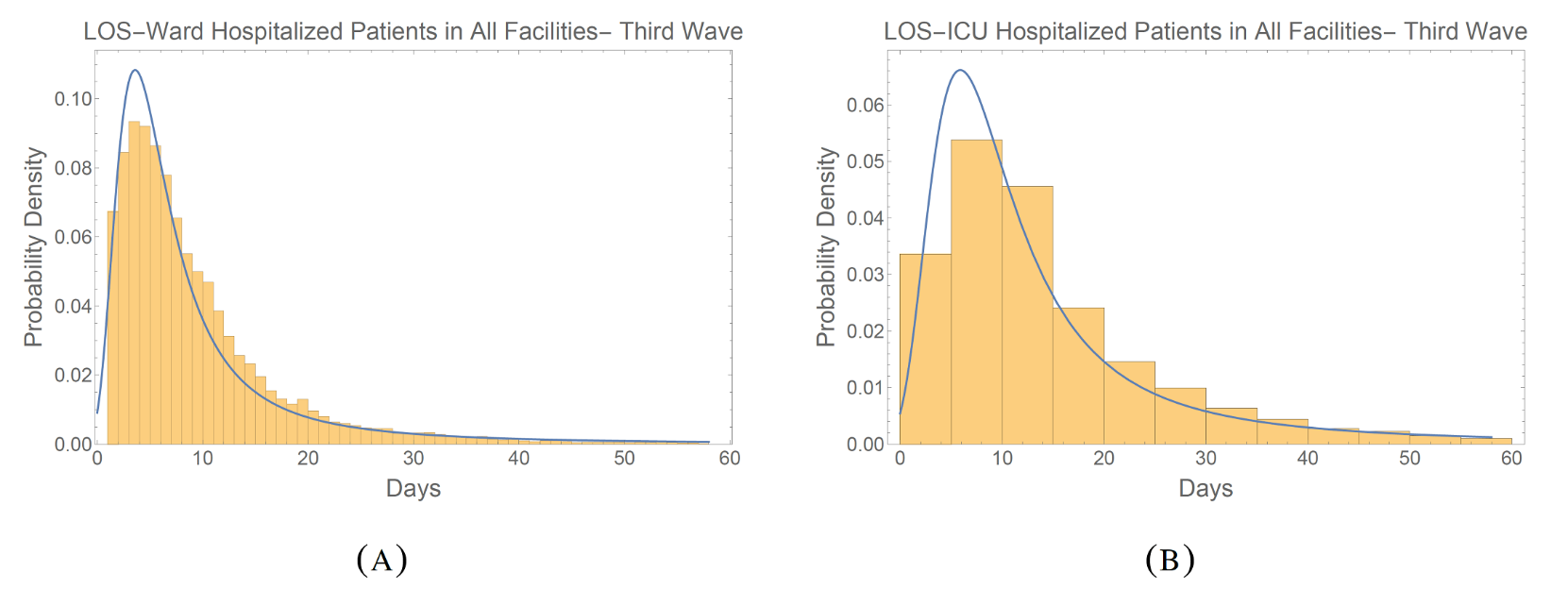


Fig G in S1 Text: Health-states flow in the community from susceptible to confirmed COVID-19 case.


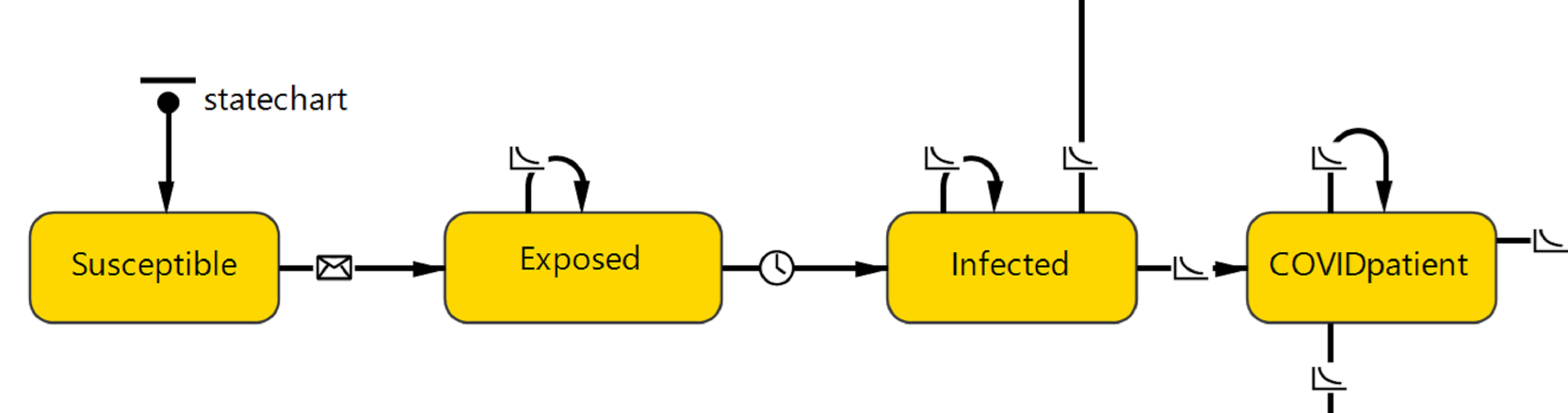


Fig H in S1 Text: Health-states flow after self-isolation


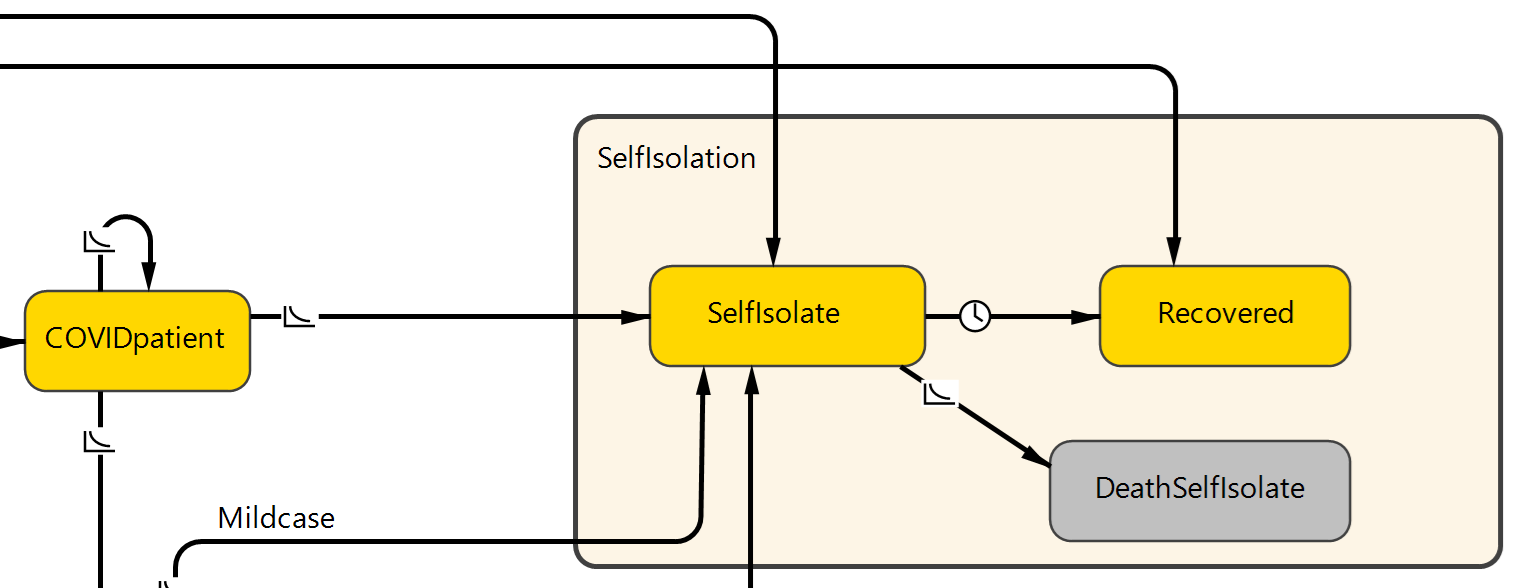


Fig I in S1 Text: In-hospital patients flow after the emergency department visit. After being confirmed as a COVID case, individuals may visit emergency department. Otherwise, they self-isolate.


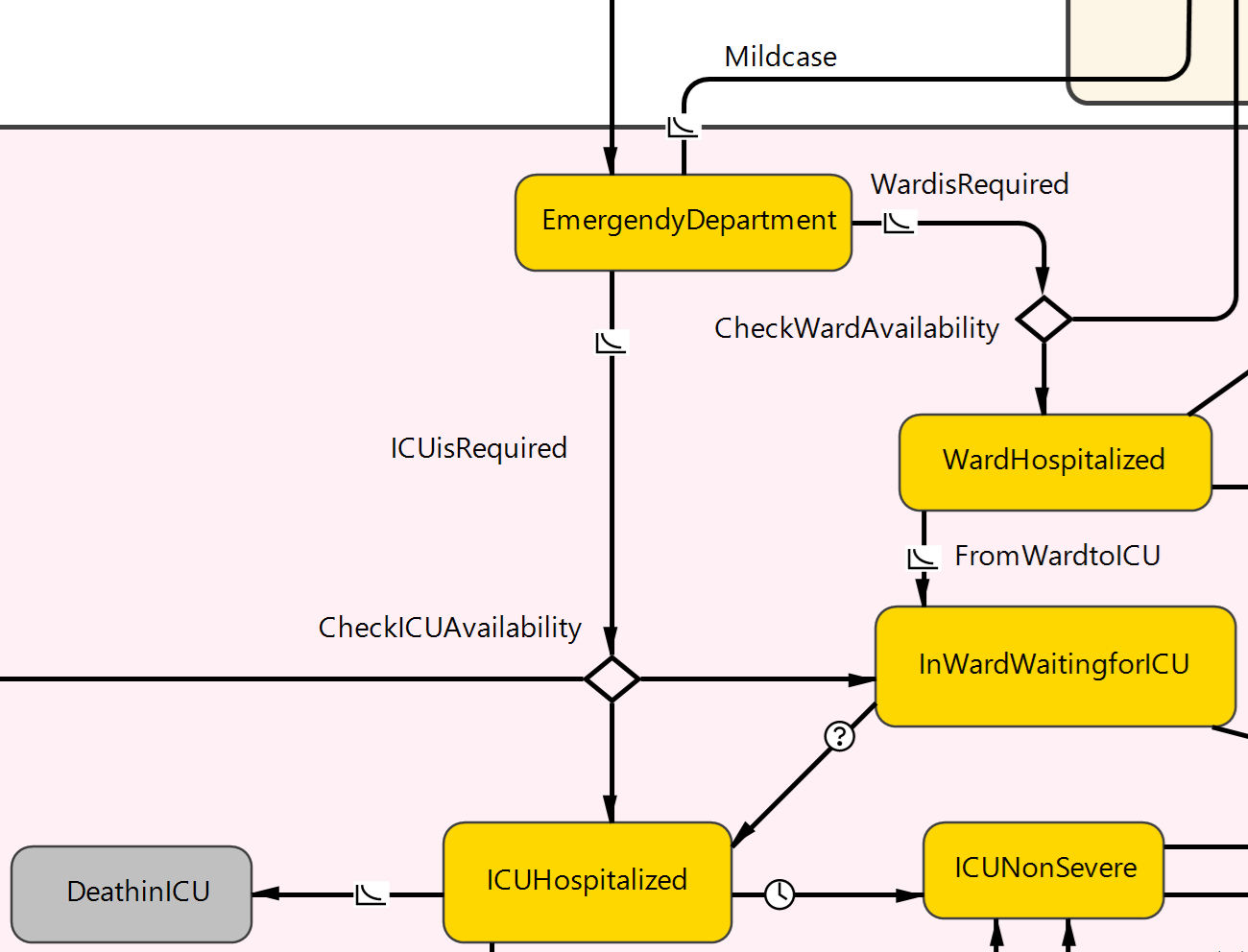


Fig J in S1 Text: Health-states in ICU. Patients may require ventilation.


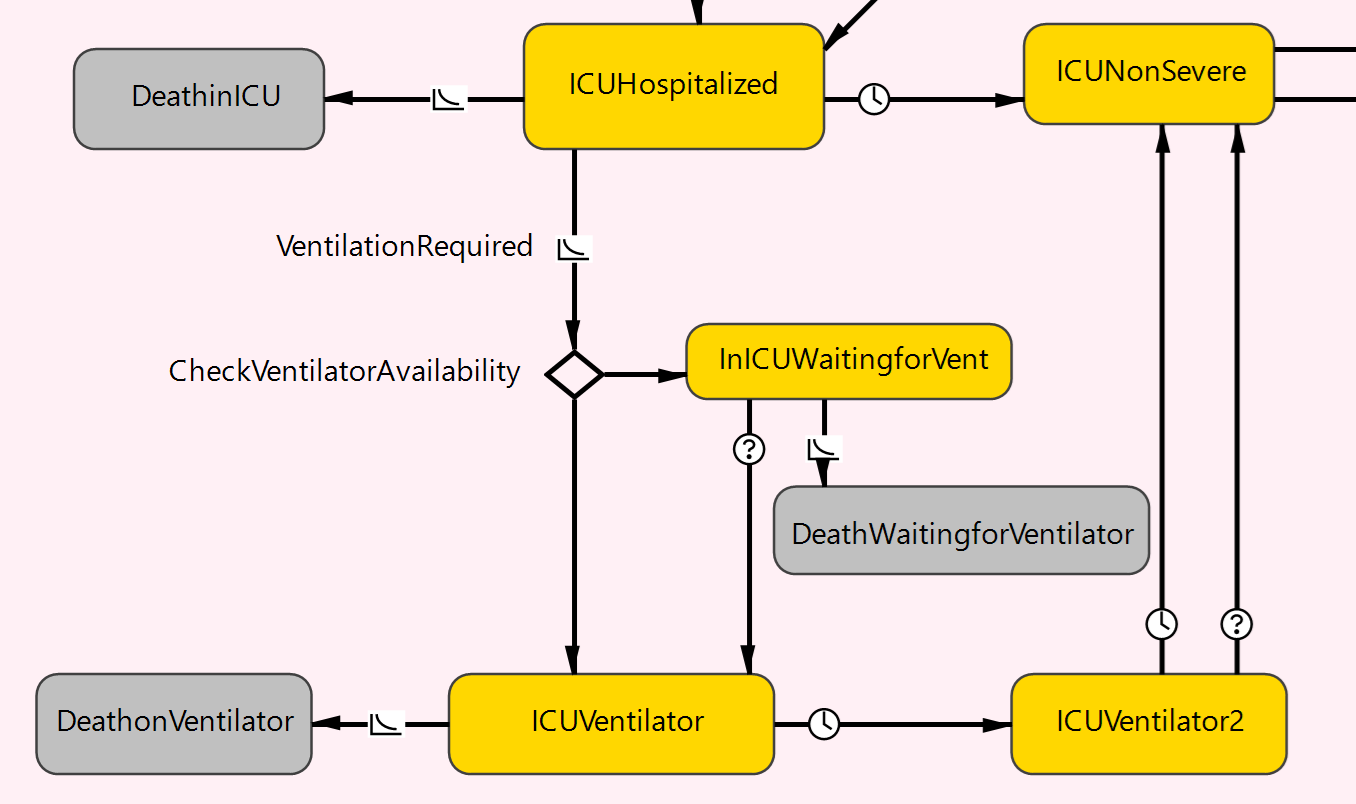


Fig K in S1 Text: Patient flow from hospitalization with severe symptoms to discharge.


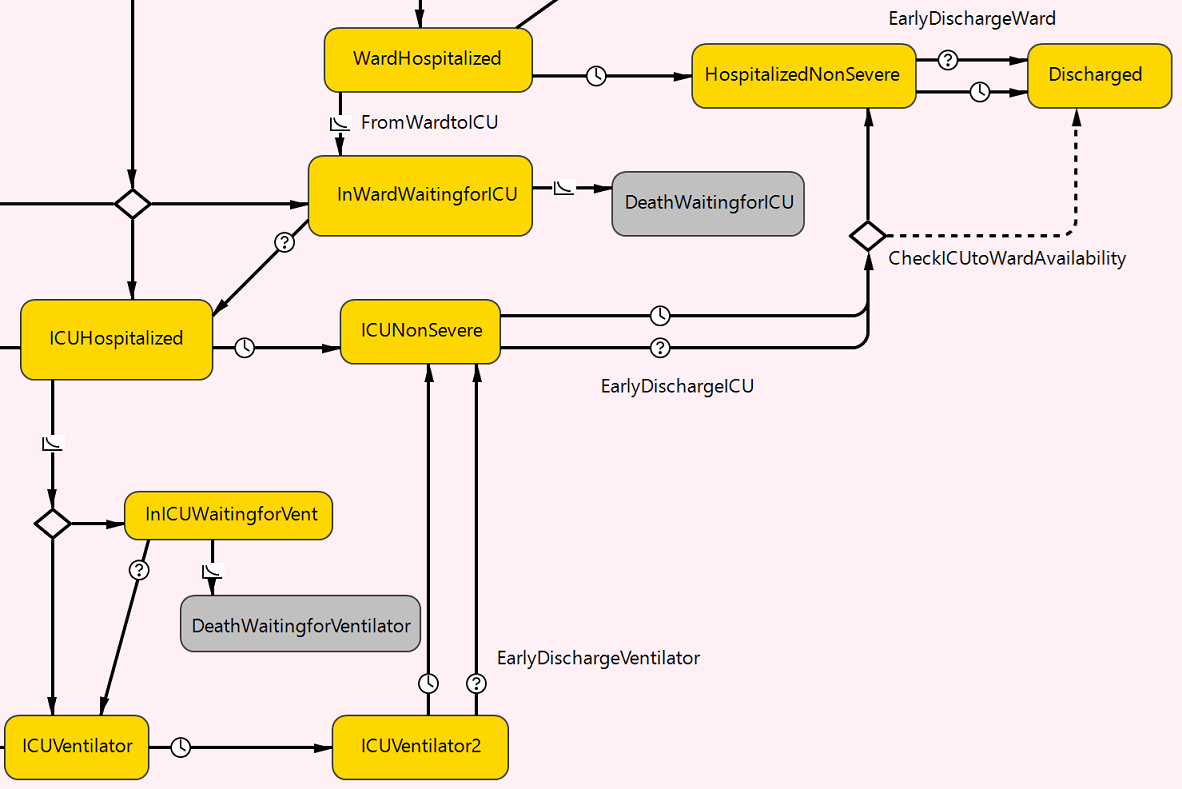


|  | Table A in S1 Text: LOS distribution fitting results |  | |
| --- | --- | --- | --- |
| Data | Landau distribution parameters  location (*µ*) | scale (*σ*) | Median |
| First wave | All 3.06 | 2.92 | 6.73 |
|  | Ward/Isolation 3 | 2.68 | 6.22 |
|  | HC/ICU 3.64 | 4.23 | 9.97 |
|  | Public 2.87 | 3.16 | 7.01 |
|  | Private 3.35 | 2.75 | 6.54 |
| Second wave | All 3.35 | 2.71 | 6.63 |
|  | Ward/Isolation 3.18 | 2.55 | 6.17 |
|  | HC/ICU 3.73 | 4.23 | 10.05 |
|  | Public 3.12 | 2.86 | 6.79 |
|  | Private 3.05 | 2.82 | 6.63 |
| Third wave | All 3.23 | 2.85 | 6.78 |
|  | Ward/Isolation 3.11 | 2.62 | 6.23 |
|  | HC/ICU 3.76 | 4.29 | 10.21 |
|  | Public 3.05 | 2.89 | 6.67 |
|  | Private 3.36 | 2.82 | 6.83 |

**Agent Based Model Formulation**

A1 The dynamics of the disease is captured by an agent-based model while keeping track of the available healthcare resources and the possibility of increasing the resources depending on the demand. An SIR-type agent-based model is considered for the disease dynamic in the popula- tion.

A2 The susceptible individuals move to the exposed state as a result of a contact with an exposed, infected or confirmed individual. The rate of transmission is different if the contact is between an exposed, infected (but not confirmed) or confirmed individual.

A3 Exposed individuals move to infected state at a given rate and a fraction of infected individuals get confirmed. Infected individuals who are not confirmed recover at a given rate.

A4 A fraction of confirmed individuals self-isolate and therefore will not transmit the disease any- more. they may recover or die (with a very low possibility). Confirmed individuals with severe symptoms visit the emergency department (ED). There are three possibilities: they may be ad- mitted to the hospital with either an ICU bed or a ward bed; if hospitalization is not required then they will be sent home for self-isolation. We assume individuals who self-isolate will not visit ED for a second time.

A5 Availability of ICU and ward bed is checked, and decisions are made based on that. For instance, if an ICU bed is needed, then we check for ICU availability: if available the patient will be

hospitalized in an ICU bed if not, he/she will be admitted to a ward bed and wait for an ICU bed. If none of these are available, the patient will be required to self-isolate.

A6 ICU patients may need a ventilator and they will be moved to a ventilator, if available. Oth- erwise, they wait for a ventilator. Previously ward hospitalized patients may need an ICU bed and will move to an ICU bed if available.

A7 State of ward and ICU bed hospitalized patients is divided into severe and non-severe and pa- tients in non-severe states can be discharged earlier than the baseline duration of hospitalization to free beds when the capacity is limited. All ICU hospitalized patients move to a ward bed before being discharged, unless the number of available ward beds is low and, in that case, they will be discharged directly from ICU. We assume patients in an ICU bed with ventilation move to an ICU bed if they do not need ventilation anymore. Patients on ventilator also have two states of severe and non-severe and they can move to an ICU bed without ventilator depending on the number of new patients in critical need for ventilation.

A8 A fixed average length of stay (ALOS) is considered for three types of resource use (ward bed, ICU and ICU with ventilation). All patients will eventually move to the non-severe state of ward hospitalization, and they will be discharged.

A9 There is a possibility of deaths for some of the states in the hospital: patients may die dur- ing a severe stage of ward bed hospitalization and severe stage of ICU and during ventilation (resource-independent probability of death while receiving the required service). Patients wait- ing for an ICU bed, or a ventilator die with a high probability (resource-dependent probability of death).

Table B in S1 Text: Parameter description and values

| Parameter | Value  first wave | second wave | third wave | Reference |
| --- | --- | --- | --- | --- |
| Simulated population size* | 5 × 105 | 5 × 105 | 8 × 105 | Assumed |
| Transmissibility reduction (exposed) | 0.5 | 0.5 | 0.4 | Assumed [[29](#_bookmark40)] |
| Contact rate reduction (confirmed) | 0.1 | 0.1 | 0.1 | Assumed |
| Infection probability | 0.28 | 0.54 | 0.51 | Model calibrated |
| Disease confirmation rate | 0.7 | 0.85 | 0.85 | Assumed |
| Self-isolation rate | 0.57 | 0.58 | 0.65 | Model calibrated |
| Duration of exposed period (days) | 5.9 | 2 | 3.5 | Model calibrated |
| Duration of illness for mild cases (days) | 11.26 | 11.11 | 11.54 | Model calibrated |
| ED visit rate | 0.43 | 0.42 | 0.35 | Model calibrated |
| Proportion of mild cases (in ED) | 0.71 | 0.63 | 0.66 | Model calibrated |
| Proportion of cases requiring a ward bed ** | 0.25 | 0.32 | 0.31 | Estimated from Data |
| Proportion of cases requiring a HC/ICU bed | 0.04 | 0.05 | 0.03 | Estimated from Data |
| Proportion of ICU patients requiring a ventilator | 0.41 | 0.53 | 0.69 | Estimated from Data |
| Average length of stay (ALOS) in a ward bed | 8.6 | 8.2 | 7.5 | Model calibrated |
| ALOS in an ICU bed | 12.9 | 9.8 | 8.7 | Model calibrated |
| ALOS on a ventilator | 4.4 | 4.8 | 4.1 | Model calibrated |
| Death rate- self-isolation*** | 1 × 10−3 | 5 × 10−3 | 5 × 10−3 | Estimated |
| Death rate- in ward | 0.014 | 0.02 | 0.036 | Model calibrated |
| Death rate- in ICU (without ventilator) | 0.02 | 0.022 | 0.045 | Model calibrated |
| Death rate on a ventilator | 0.07 | 0.074 | 0.08 | Model calibrated |
| Death rate- waiting for ICU | 0.25 | 0.25 | 0.25 | Assumed |
| Death rate- waiting for a ventilator | 0.3 | 0.3 | 0.3 | Assumed |
| Maximum ward bed available (all hospitals) | [7000,10000] |  |  | Assumed |
| Maximum ICU bed available (all hospitals) | [2000,4000] |  |  | Assumed |
| Maximum ventilators available (all hospitals) | [800,1200] |  |  | Assumed |

* A small population size was used to generate the epidemic curves and the daily hospitalizations according to the data form the Gauteng province. ** ED visits are divided into two groups: those requiring a general/isolation ward bed and not HC/ICU and those requiring a HC/IC bed. *** The average case fatality rate in SA is 3%. We estimated the death rate based on this value.
